# Review on Epidemiology and Public Health Importance of Goat Tuberculosis in Ethiopia

**DOI:** 10.1155/2020/8898874

**Published:** 2020-09-25

**Authors:** Zelalem Ayana Dibessa

**Affiliations:** School of Veterinary Medicine College of Agricultural Science, Bule Hora University, P.O. Box 144, Bule Hora, Ethiopia

## Abstract

Small ruminant is an important and integral part of livestock in Ethiopia. Especially, goats are attractive to people of Ethiopia because of the ability to resist challenges, easily adapt to different ecological regions, and need small land to rearing and small initial capital in which poor people can engaged in the production system. In spite of the presence of large number of goat population in Ethiopia, it fails to utilize expected productivity due to many factors. Among the factors, which limit the economic returns of goats, diseases stand frontline. Tuberculosis is one the diseases that affect goats' health and production in Ethiopia. Goat tuberculosis is a chronic disease, which is characterized by the development of granulomas, essentially in the respiratory tract and related lymph nodes, from which the mycobacteria are discharged and contaminate other susceptible animals. Goat tuberculosis has a public health implication in Ethiopia because of the farmers' habit of consuming raw goat milk and its products, and they do have consistent or day-to-day contact with their goats. The etiological agents also transmitted to humans through the aerogenous route from those animals with active cases in the herd. The infection has been reported from several parts of different areas of the country dependent on the abattoir inspections. Therefore, attention should be given towards the control of tuberculosis in livestock; public health education on zoonotic importance of the disease or awareness creation and the national tuberculosis control needs to consider the one health approach, and further epidemiological studies should be undertaken.

## 1. Background

Livestock is an important and integral component of agriculture, which is the pillar of the Ethiopian economy. Ethiopia is believed to have the largest livestock population in Africa, which comprises 60.3 million cattle, 31.3 million sheep, and 32.7 million goat populations, and are widely distributed across the different agroecological zones of the country [1].

Goats are a significant domesticated animals segment in many parts of the world including Ethiopia. They become attractive due to the ability to resist different difficulties or harsh ecological conditions. Because of this reason, many poor rural areas of different countries rear goats [2]. On a morphological basis, goat species has ability to adopt to different climatic conditions more promptly than other ruminant species, and thus, they keep on being a significant source of income and sustenance to many poor and marginal farmers around the world [3]. Camels and goats have consistently been viewed as extremely valuable animals for their great items and simplicity in dealing and they do not compute man for food and eat small feeds. Small ruminants, particularly goats, are widely produced on degraded land, shrub land, and timberland territories where different species cannot endure [4]. This protects the rural areas from erosion and degradation and adds to keep of good agroenvironmental practices. They additionally tolerate mortality during times of dry season than large ruminants [5, 6].

In Ethiopia, small ruminants serve different business works by providing food and sustenance, income, and raw material for industries. They additionally fill different significant social needs, for example, wedding blessings, gift for poor family members, and inheritance. Women have a great deal of command over small ruminants contrasted with other domesticated animal species and closely involved in small ruminant health management. The animals regularly fill in as crisis wellsprings of assets for family unit commitments and individual use. The increase of consumption of small ruminant meat items both locally and universally offers an open door for small ruminant managers to get to better markets [7].

In spite of the presence of a large number of small ruminant population in Ethiopia, it fails to exploit the expected productivity due to many factors. Among the many factors, which limit these economic returns from small ruminants, are diseases that stand in the front line. One of those diseases that affect small ruminants is tuberculosis.

Tuberculosis is a chronic disease which has a zoonotic importance and hampers the productivity of goat in many parts of Ethiopia and reported by some authors such as Deresa et al. [8]. Tuberculosis is a disease which circulates between humans and animals. Thus, it has a serious zoonotic and reverse zoonotic importance. Ethiopia had also confirmed transmission of *M. tuberculosis* from farmers to their cattle, goat, and camel [9].

Therefore, there is an urgent need to advocate for effective control measures because in Ethiopia, there are more number of people who have small ruminants and their life depends on them, with a habit of having close physical contact and sharing the same dwelling with their animals, and they consume raw animal products such as milk and meat. This creates a potential public health concern for transmission of zoonotic diseases such as tuberculosis. So that, determination of prevalence of goat tuberculosis and other animals is the first step to appraising the disease transmission risk and burden. Even though goat tuberculosis has long been reported, in different areas, there is no sufficient information on the epidemiology and public health importance in Ethiopia at the national level. Therefore, the objectives of this review paper are to compile the available information on the epidemiology of goat tuberculosis, to indicate the zoonotic importance of goat tuberculosis, and to highlight prevention methods of goat tuberculosis as the basis for policy makers for effective control strategies.

## 2. Search Strategy

This critical review is based on a search in PubMed and PMC (https://http://www.google.de/search) using the terms goat, tuberculosis, epidemiology, public health importance, and Ethiopia. The title and abstract of the obtained hits were evaluated, and articles referring to the goat tuberculosis and public health importance were studied in detail. In addition, our own archives were used as a source of additional information.

## 3. Review

### 3.1. Goat (Caprine) Tuberculosis

#### 3.1.1. Etiology of the Diseases

TB is a chronic, primarily respiratory infectious disease of mammals caused by the *Mycobacterium tuberculosis* (MTB) complex, a group of closely related bacteria that include *M. bovis* responsible for TB in cattle and other mammals, including, occasionally, people. Cattle are the natural hosts of *M. bovis*, but nearly all warm-blooded animals, including goats, deer, pigs, camelids, and badgers, are susceptible to the infection. This broad range of animal hosts complicates the eradication of tuberculosis in different animal species. Goats are also susceptible to *M. tuberculosi*s, the primary cause of TB in people, *M. avium* and *M. Kansasii*, but these rarely cause caprine *TB*. Primarily, a goat affected by the *M. tuberculosis* complex, from the *M. tuberculosis* complex *M. caprae*, is the principal one [10].

Tuberculosis in goat and sheep is caused by members of the *Mycobacterium tuberculosis* complex, mostly *Mycobacterium bovis* and *Mycobacterium caprae* and, in some cases, by *Mycobacterium tuberculosis* [11].

#### 3.1.2. Epidemiology of Goat Tuberculosis

TB can spread either directly (animal-to-animal), particularly by aerosol, or indirectly (via infective material, for example, manure, urine, bedding, contaminated feed, and water). The organisms can survive for long periods in moist conditions or organic matter. Spread from the dam to kid through the feeding of milk from animals with TB of the udder is also possible. Goats also act as “amplifier hosts,” that is, they have the ability to spread the disease to other goats, as well as other species (including humans), cohabiting with them. Once the infection is established within a herd, it appears to spread rapidly within the herd [10].

Goats may play a role in the conservation and transmission of tuberculosis. Lack of knowledge of the disease and lack of testing (control programs and premovement tests) may result in the dissemination of the infection to other flocks and to other animal species. The cross infection of *M*. *bovis* between cattle and goats has been reported [11].

Epidemiological studies have indicated that tuberculosis in goat and sheep has a wide global distribution, being reported in several countries of the world including New Zealand, Sudan, Spain, Nigeria, the United Kingdom, Italy, Algeria, and Ethiopia [12]. Caprine tuberculosis poses a risk to goat health and production in the developing world. There has been a recent increase in caprine tuberculosis in several countries, even in those practicing a long-standing test-and-slaughter policy [11]. It is reported that the infection is widespread in Africa where goats cograze with cattle that were not subjected to tuberculosis testing and slaughter protocols [13].

#### 3.1.3. Source of Infection and Mode of Transmission

Goats are quite susceptible, and if they are kept in association with infected herds of cattle, the incidence may be as high as 70%. Transmission of tuberculosis, generally, is through the respiratory tract. Infectious organisms can be found in respiratory secretions, feces, milk, urine, vaginal secretions, semen, and draining lymph nodes. Once the tuberculosis bacillus enters through the respiratory tract, it invades the local lymph nodes and causes granuloma formation with central necrosis of the lymph node. Occasionally abdominal involvement is observed, suggesting that ingestion may be a possible route of transmission [14].

Risk factors such as herd size, keeping herd with other livestock species, contact with other herds, annual migration dynamics, the recent introduction of new animals to the herd, and other risk factors could be associated with herd positivity to tuberculosis [9].

In Ethiopia, mixed farming of cattle and goats is a common practice. Livestock move freely from one region to another and from farm-to-farm. Thus, this practice poses a high risk of inter- and interspecies transmission and spread of *M. bovis* infection. This mixed farming of small and large ruminants is especially a risk to goats in countries such as Ethiopia where bovine TB is endemic [15].

Caprine tuberculosis poses a risk to goat health and production in the developing world. There has been a recent increase in caprine tuberculosis in several countries, even in those practicing a long-standing test-and-slaughter policy [11].

Despite continuous close contact and sharing of pastures with potentially infected cattle, the prevalence based on a single intradermal cervical comparative tuberculin (SICCT) test in small ruminants was very low [16]. Because of this a reason, the goats serve as a *reservoir* for a long period of time and transmitted diseases to other susceptible animals.

In goats, the disease normally spread through head-to-head contact, which will include sharing of contaminated feed source and water plates, as well as infected aerosols spread from breath. Tuberculosis can affect the udder, in which case the milk is infective until or unless it is pasteurized. Infected sputum coughed up can be swallowed and, thus, infect the gastrointestinal tract. Most commonly in goats, the cough is usually seen as a chronic cough, which is unresponsive to treatment and may be accompanied by gradual loss of weight and, sometimes, diarrhoea [13]. The predilection site for tuberculosis in goats is the lower respiratory tract and the associated lymph nodes [17].

Infected goat herds can constitute a *reservoir* of TB inducing mycobacteria in the field, posing a risk of infection to cattle and wildlife [18, 19]. Furthermore, *caprine* TB not only may hamper the eradication campaigns against bovine TB in affected areas but also responsible for cases of TB in humans [20].

#### 3.1.4. Pathogenesis

Tuberculosis spreads in the body by two stages, the primary complex and post primary dissemination. The primary complex consists of the lesion at the point of entry and in the local lymph node. A lesion at the point of entry is common when infection is by inhalation. When infection occurs via the alimentary tract, a lesion at the site of entry is unusual, although tonsillar and intestinal ulcers may occur. More commonly, the only observable lesion is in the pharyngeal or mesenteric lymph nodes. A visible primary focus develops within 8 days of entry being affected by the bacteria. Calcification of the lesions commences about 2 weeks later. The developing necrotic focus is soon surrounded by granulation tissue, monocytes, and plasma cells and the pathognomonic “tubercle” is established. Bacteria pass from this primary focus, which is in the respiratory tract 90–95%, to a regional lymph node and cause the development of a similar lesion there. Postprimary dissemination from the primary complex may take the form of acute tuberculosis, discrete nodular lesions in various organs, or chronic organ tuberculosis caused by endogenous or exogenous reinfection of tissues rendered allergic to tuberculo-protein. In the latter case, there may be no involvement of the local lymph node. Depending upon the sites of localization of infection, clinical signs vary, but because the disease is always progressive, there is the constant underlying toxaemia that causes weakness, debility, and the eventual death of the animal. In cattle, horses, sheep, and goats, the disease is progressive, and although generalized, tuberculosis is not uncommon [10].

#### 3.1.5. Clinical Signs

Clinical signs of tuberculosis include weight loss and mild respiratory signs. Early in the course of the disease, affected animals exhibit a deep, moist-sounding, chronic cough. As the disease progresses, tachypnea, dyspnea, and abnormal lung sounds develop [10].

The involvement of the respiratory tract in tuberculous goats renders them suitable to act as a maintenance host for bovine TB and to disseminate the disease [21, 22]. TB caused by *M. caprae* is known to spread from infected flocks to in-contact cattle herds [21].

Clinical signs are nonspecific, making a diagnosis of TB difficult. TB should be considered in cases of chronic loss of condition and appetite, reduced milk yield, and debilitating disease, with or without respiratory signs. A chronic cough can be a sign of TB in goats and should, in particular, be considered when a goat has failed to respond to antibiotic treatment for a respiratory infection. Goats may have extensive lesions without obvious clinical signs and occur as a disseminated disease involving the thorax (mediastinal lymph nodes, lungs, and pleura) and abdomen (peritoneum, liver, spleen, and mesenteric lymph nodes); occasionally, superficial lymph nodes are enlarged and palpable [10]. The lymph nodes are also encapsulated and contain yellow to orange, creamy to caseous purulent material and gritty foci. Respiratory lymph nodes are affected more frequently than the liver or mesenteric lymph nodes. Histopathologic findings include the presence of acid-fast organisms and central calcification and caseation surrounded by epithelial cells and fibrosis [23].

Bronchopneumonia is the known form of the disease in these species and is manifested by cough and terminal dyspnoea. In some goats, intestinal ulceration, diarrhoea, and enlargement of the lymph nodes of the alimentary tract occur. In goats, the disease is only slowly progressive, and in affected flocks, many more reactors and necropsy-positive cases are often found than would be expected from the clinical cases, which are evident. In kids, the disease may be more rapidly progressive and cause early death [24].

At postmortem findings, tuberculosis granulomas in one or more lymph nodes and caseous tubercles in the lung, liver, or spleen were seen. The lungs and associated lymph nodes were the sites in which caseous tubercles were most frequently seen. Although tubercle production is classically described in goats as in cattle, these cases often did not develop tubercle lesions in the lungs, producing instead large abscesses with liquid white or cream pus, which is often quickly eroded into the airways, resulting in a greater tendency for disease spread by aerosol. Lung lesions were white or cream, multifocal, and often occurred in areas of purple consolidation. Bronchial and mediastinal lymph nodes contained lesions varying from pinpoint foci to large caseous lesions with mineralization. Lesions were also present in the mesenteric and retropharyngeal lymph nodes [10].

#### 3.1.6. Pathology

The course of disease, localization of lesions, and appearance of macroscopic lesions are similar to those described in cattle; however, in most animals, they are widespread. Common necropsy findings reveal granulomatous/caseous/caseocalcareous lesions of various sizes in the respiratory tract (lung and thoracic lymph nodes), pleura, liver, and mesenteric lymph nodes. Typical caseous lesions in lymph nodes range from pinpoint foci to large lesions [17, 25]. *Caprine* and *bovine* TB are closely related in regard to the immune response and pathological characteristics [26]. In normal contaminations, TB in goats and sheep, as in dairy cattle, is fundamentally an interminable disease that causes exudative granulomatous caseous intense injuries in the lungs and related lymph nodes.

Occasionally, tuberculous lesions may also be found in the upper respiratory tract lymph nodes and other organs, such as the spleen, liver, or mesenteric lymph nodes [17, 27]. Goats show a solid disposition to create liquefactive necrosis and natural hollows inside tuberculous granulomas that is surprisingly like those seen in human TB. Histologically, the lesions are similar to those observed in cattle and humans. Typical tuberculous granulomatous necrotizing lesions are observed, characterized by central caseous necrosis, often with some mineralisation, surrounded by macrophages, foamy macrophages, numerous giant cells, lymphocytes, and a fibrotic capsule. Acid-fast bacilli are usually present inside the caseous necrosis in very low numbers [26].

#### 3.1.7. Diagnostic Tests

The diagnosis of caprine tuberculosis is similar to BTB diagnosis with minor modification. The tuberculin skin test or the gamma interferon (IFNᵞ) assay can be also applied, with minor modifications, for diagnosis of TB in goats [25]. Clinical signs determination, for example, weight loss and mild respiratory signs such as exhibition of a deep, moist-sounding, chronic cough, tachypnea, dyspnea, and abnormal lung sounds. Loss of milk production and respiratory pain signs are vague and might be used as methods for diagnosis [17, 28]. In addition, postmortem examination, single intradermal comparative cervical tuberculin (SICCT), and Enferplex ELISA (SureFarm) are tests used to diagnose tuberculosis in goats [10, 29, 30].

## 4. Distribution of Goat Tuberculosis in Ethiopia

Ethiopia has high incidence rate of TB infection, and the disease is one of the major public health problems in the country. In Ethiopia, as many researchers' report, tuberculosis is distributed in different regions (Table 1).

## 5. Control and Prevention of Goat Tuberculosis

Various issues hamper progress in the prevention and control of zoonotic TB in Ethiopia. Anyhow, the general feeling that it is anything but an issue in Africa, the absence of satisfactory infection control and destruction approaches, the absence of continental reference databases and research facility limit with respect to the isolation and genotyping of *M. bovis*, and the weak transdisciplinary and interlaboratory collaboration are significant obstructions that should be overcome to get some level of participation in regional and continental levels [39].

Successfully eradicating the disease is dependent on the accessibility and operation of distinguishing all the infected animals. In Africa, including Ethiopia, the absence of finance generally decides the indicative strategies that can be applied, and inspection at slaughter remains the main reasonable strategy by which the infection can be analysed and its spread and prevalence can be decided. In many African countries, only a small percentage of carcasses for human consumption are inspected, and most domesticated animals are slaughtered informally and are not examined by meat inspectors (professionals) [10, 39].

Currently, there is no a national tuberculosis control or anticipation program in Ethiopia because of principally inadequate finance, infrastructure, and human resources. An absence of awareness, by farmers and traders of a risk-based marketing system or stay away from the risk of contamination with BTB, is likewise universal and ought to be tended to while performing future control programs [8].

Another issue that ought to be corrected is the current unrestricted movements of infected animals, since contact with other domesticated animals or wild life comprises a significant manner by which the disease is distributed. There is no limitation of movement of animals throughout the country. This makes difficult to control TB and different diseases likewise.

In the country, there is no test-and-slaughter policy, no animals' movement restriction, and no animal identification and a tracking system, and in general, absence of information and awareness about isolation and quarantine practices are a portion of the difficulties that make the future prevention and control of tuberculosis in Ethiopia. Even up-to-date, except some export abattoirs, the majority of the people slaughter the small ruminants without any antemortem and postmortem examination and slaughter in open space, whether that animal has diseases or not. Also, there is scarcity of small ruminant abattoirs in the country, when we compare with large remnants abattoirs.

In goats, culling of the infected one and avoidance of the mixed farming system with infected cattle help to minimize the occurrence of tuberculosis in goats. Pasteurization of milk, thorough inspection of meat, and condemnation of the affected part are some techniques to reduce the risk of tuberculosis in humans. In rural areas, sharing of the common shelter is one risk factor for human tuberculosis. So, avoidance of this characteristic helps to reduce zoonotic tuberculosis in humans.

## 6. Public Health Significance of Goat Tuberculosis

The transmission of TB from goats to people can occur in different ways such as consumption of raw (unpasteurized) milk or dairy products made with unpasteurized milk from goats infected with tuberculosis (effective pasteurization removes the risk of transmission of TB to humans). By inhaling, the bacteria are shed by infectious animals in respiratory and other secretions. The other is through contamination of unprotected cuts or abrasions in the skin while handling infected animals or their carcasses [10].

**Figure 1:**
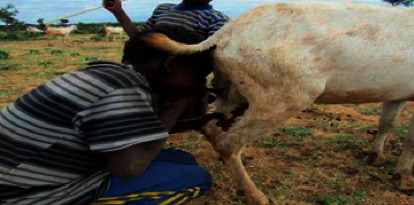
Risky milk consumption behaviour.

In rural parts of Ethiopia, the life of people is mainly dependent on animals and their products, and the relationship with them is very close. Moreover, people often consume raw animal products [40]. According to Golo et al. [41], about 98.88% of the people of Yabelo district consume sheep and goat milk. Out of this percent, 25 (28.4%) replied they consumed raw products which predispose to zoonotic diseases. Raw milk consumption is very common in Borana pastoral areas. It was observed that people buy raw milk and consume it on the spot during market days and, sometimes, children consume goat milk directly from the udder (Figure 1). Other practices observed that could potentially impair the health of consumers include selling raw milk for direct consumption on market days without heat treatment [42].

Milk consumption directly from the udder of a goat by children in Borana (a common practice during herding; source: [42]).

## 7. Conclusions and Recommendations

Tuberculosis is endemic in humans and animals in Ethiopia. It is well studied on bovine, but the attention is not given to goat tuberculosis. Goats are sensitive to tuberculosis. Because of the vague of clinical signs of tuberculosis on goat, they highly disseminate the diseases to man and other species of animals. Goats harbour the diseases from infected animals at daytime by close contact while they are kept mixed with cattle and, then, at night time, they will transmit to humans by sharing common shelter, especially in rural areas of Ethiopia. So, the goat has high influence in epidemiology and public health importance of tuberculosis. Because of this reason, knowing the prevalence and epidemiology is the key role in controlling tuberculosis in goat and, furthermore, in different hosts. The people who rear the goat in Ethiopia have no awareness of zoonotic diseases; rather, than they think only about the medicinal value of goat products.

In line with the abovementioned conclusive remarks, the following recommendations are forwarded:

  Public health awareness must be launched and needed to raise community awareness about the risk of goat tuberculosis transmission through sharing common shelters and consumption of raw animal products; and routes of zoonosis are of extreme importance for effective implementation of TB control measures.
  Collaboration and communication across different sectors and disciplines must be established to control tuberculosis. The most relevant sectors which include healthcare services, veterinary services, food safety authorities, wildlife authorities, farming organizations, consumer groups, trade organizations, educational bodies, and financial institutions should be involved.
  Policies and guidelines must be developed and implemented for the prevention, surveillance, diagnosis, and treatment of zoonotic TB, in line with intergovernmental standards where applicable.
  Mixed farming of cattle and goats must be prohibited. It is known that tuberculosis is highly prevalent in cattle, so if a goat stays with infected cattle, it will be infected because of being sensitive to tuberculosis and amplifies the disease.
  Restriction of movement of the animals must be implemented. Restriction of livestock to move freely from one region to another and from farm-to-farm should be implemented. Thus, this practice poses a high risk of inter- and interspecies transmission and spread of tuberculosis infection.
  Test-and-slaughter policy must be practiced, and culling of infected animals is an activity that reduces the prevalence of goat tuberculosis and minimizes the transfer of diseases to human.

## Figures and Tables

**Table 1:** Prevalence of some goat tuberculosis in different regions of Ethiopia.

Study region	Study areas/the place where the sample is drawn	Year of investigation	No. animals/carcasses examined	Overall prevalence	References
Oromia	Borena	2013	472	23 (4.9)	[8]
	Arsi	2013	360	13 (3.6)	[8]
	Jimma	2013	386	15 (3.9)	[8]
	Modjo Modern Export Abattoir	2003	1,536	65(4.2%)	[13]
	Modjo Modem Export Abattoir	1996	1,536	65(4.2%)	[31]
	Modjo Modern Export Abattoir	2012	1,536	65 (4.2%)	[32]
	Modjo Modern Export Abattoir	2010	1,536	65 (4.2%)	[33]
	Helimex Export Abattoir, Debre-Zeit,	2005	1,152	50(4.3%)	[34]
	Adami Tulu Jiddo Kombolcha district, East Showa zone	2011	630	20 (3.1%)	[35]
Amahara	South Wello	2013	386	10 (2.6)	[8]
Tigary	Kola Tembein district		300	7 (2.33%)	[36]
Afar	Chifra district.	2012	320	20(6.5%)	[37]
Somali	From different districts of Somale	2013	386	8(2.1)	[8]
	Filtu woreda of Liben zone	2013	517	10(1.9%)	[9]
Southern Nations, Nationalities, and Peoples Region (SNNPR)	Hamer	2010	186	0	[38]
	Meskan district of Gurage zone	2011	63	1(1.56%).	[16]

## Data Availability

All data generated or analysed during this study are included in this published article and its supplementary information files.
